# Comparison between Colony Morphology and Molecular Phylogeny in the Caribbean Scleractinian Coral Genus *Madracis*

**DOI:** 10.1371/journal.pone.0071287

**Published:** 2013-08-14

**Authors:** Maxim V. Filatov, Pedro R. Frade, Rolf P. M. Bak, Mark J. A. Vermeij, Jaap A. Kaandorp

**Affiliations:** 1 Section Computational Science, Faculty of Science, University of Amsterdam, Amsterdam, The Netherlands; 2 Department of Marine Biology, University of Vienna, Vienna, Austria; 3 Royal Netherlands Institute of Sea Research, Den Burg, The Netherlands; 4 Caribbean Research and Management of Biodiversity (CARMABI) Foundation, Curaçao, Netherlands Antilles; 5 Aquatic Microbiology, Institute for Biodiversity and Ecosystem Dynamics, University of Amsterdam, Amsterdam, The Netherlands; King Abdullah University of Science and Technology, Saudi Arabia

## Abstract

A major challenge in coral biology is to find the most adequate and phylogenetically informative characters that allow for distinction of closely related coral species. Therefore, data on corallite morphology and genetic data are often combined to increase phylogenetic resolution. In this study, we address the question to which degree genetic data and quantitative information on overall coral colony morphologies identify similar groupings within closely related morphospecies of the Caribbean coral genus *Madracis*. Such comparison of phylogenies based on colony morphology and genetic data will also provide insight into the degree to which genotype and phenotype overlap. We have measured morphological features of three closely related Caribbean coral species of the genus *Madracis* (*M. formosa*, *M. decactis* and *M. carmabi*). Morphological differences were then compared with phylogenies of the same species based on two nuclear DNA markers, i.e. ATPSα and SRP54. Our analysis showed that phylogenetic trees based on (macroscopical) morphological properties and phylogenetic trees based on DNA markers ATPSα and SRP54 are partially similar indicating that morphological characteristics at the colony level provide another axis, in addition to commonly used features such as corallite morphology and ecological information, to delineate genetically different coral species. We discuss this new method that allows systematic quantitative comparison between morphological characteristics of entire colonies and genetic data.

## Introduction

Classical morphological taxonomy of corals is generally based on detailed descriptions of corallite characteristics [1]. In contrast, overall colony morphology characteristics are often described in a very qualitative and informal way. Given the large variation in coral colony morphologies [2] quantitative methods that allow for accurate measurement and quantification of such variation would improve our ability to use such data for increased taxonomic resolution in studies on coral systematics. Existing morphometrical techniques to describe whole colony or organismal morphology are often based on landmark-based techniques [3] that are foremost useful in unitary organisms with a well-defined body plan. Very few methods exist to quantify and compare complex-shaped biological objects.

A recent review [4] of successful morphological phylogenetic studies on corals showed that coral morphology is most often described using skeletal characteristics, such as corallite, septal or skeletal structure. Because precisely measured morphological traits are difficult to obtain for three dimensional branching structures (e.g., branching angle and branch spacing), rigorous quantitative morphological descriptions at the colony level are generally impossible to produce. Another difficulty with morphological data is quantification. Skeletal characteristics are usually encoded at discrete intervals using a “character matrix” [5], [6]. However, morphological characters of whole coral colonies are often continuous in nature, which makes assigning discrete values to them (e.g. a colony shape is encoded as 0-massive, 1-encrusting etc.) [6] subjective and unrepresentative of the true variation that must be quantified. Crucial information required to resolve differences among species could hence be lost and intermediate morphologies could be assigned incorrectly. Therefore, exact and continuous measurements of three dimensional morphological are expected to be more informative [7] and increase statistical resolution.

Morphological characteristics at the colony level can be precisely measured using newly developed CT-scanning techniques [8]. For example, morphological analysis of three-dimensional images obtained with Computer Tomography (CT) allowed for the correct assignment of 75% of the morphospecies that comprise the Caribbean coral genus *Madracis* as identified based on traditionally used skeletal features [9].

Molecular evidence reveals that traditional, morphology-based, phylogenies of many coral taxa are not well resolved [6]. Morphological analyses of skeletal characteristics rarely yield the same clades of coral species when compared to molecular phylogenetic trees [10], [11], [12]. Genetically determined clusters often consist of more than one (morpho) species, which can be explained through phenotypic plasticity, morphological convergence (homoplasy), recent speciation with incomplete lineage sorting, morphological stasis, improper delineation of species boundaries or interspecific introgressive hybridization [13], [14], [15], [16]. Irrespective of the occurrence of such phenomena that disturb the correlation between genetic and morphological distances, having a richer morphological classification system is certainly of advantage. The higher the variation contained by the analyzed morphological parameter, the stronger is the classification power of distinct morphological lineages, and therefore the lower the overlap between distinct shapes. Continuous morphological data derived from whole colony characteristics could increase resolution of coral phylogenies when combined with genetically based species assignments.

**Figure 1:**
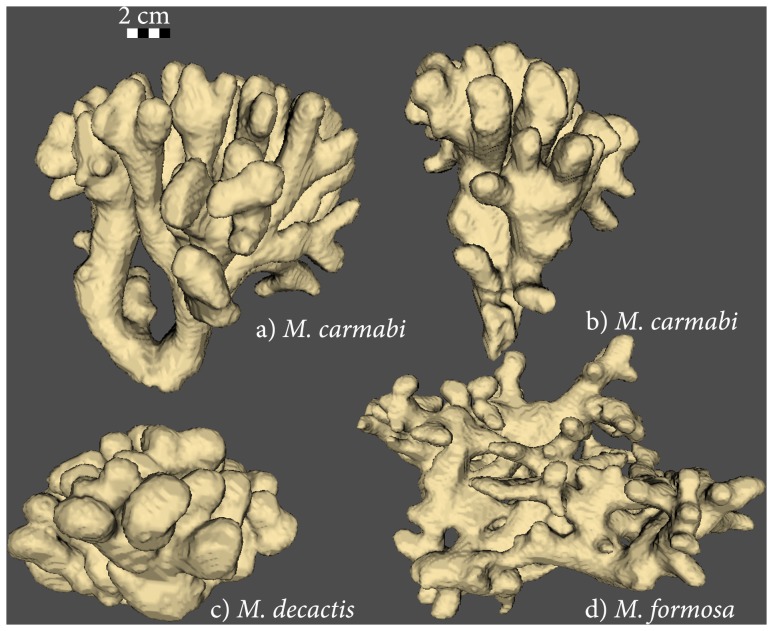
CT-scans of four Madracis colonies. Volume rendering of the CT-scans of the Madracis coral colonies: a) *M. carmabi*, b) *M. carmabi* sample Car436 in the data set, c) *M. decactis* and d) *M. formosa.*

The goal of our study is to, for the first time, quantify to what degree differences in coral colony morphology correspond to genetic differences of the same coral colonies. To achieve this goal we compared genetic differences (based on the markers ATPSα and SRP54) with measurements of 3D colony characteristics, i.e. branch thickness, branch spacing and branch length to thickness ratio; see [9], for three *Madracis* species that are not only closely related genetically, but that also share a similar branching colony morphology (figure 1). Our findings show that classification of the coral colonies based on overall colony morphology separates the data set (with few exceptions) into three groups. This classification corresponds partially with classification based on genetic differences among the same species. Further, we have also found an indication for the hybrid origin of *M. carmabi* that is likely a hybrid species between *M. formosa* and *M. decactis* [17].

## Results

**Figure 2:**
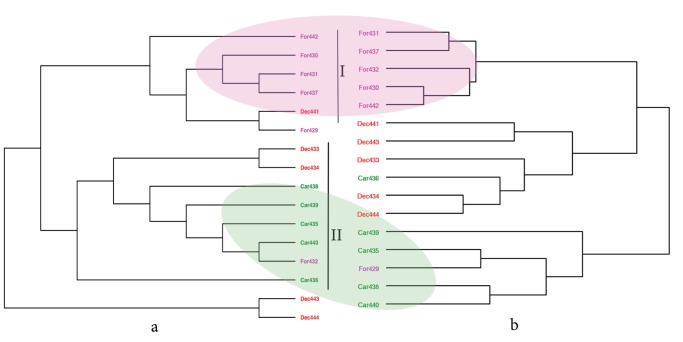
Comparison between genetic and morphological clustering. a) Topology of the phylogenetic tree inferred from average ATPSα genetic distances using maximum likelihood method. b) Morphological tree inferred from the three main morphological features (branch thickness, branch spacing and branch length to thickness ratio) using average linkage. Species codes represent the species names (Car - M. carmabi, For – M. formosa, Dec – M. decactis) followed by a sample number. Coloured ellipses indicate similar clades in both trees.

**Figure 3:**
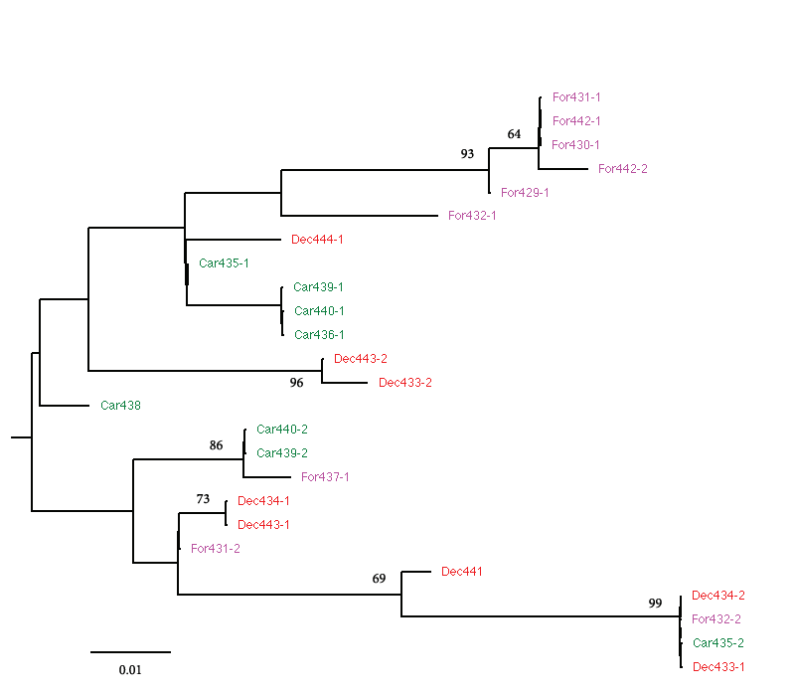
Maximum likelihood tree. Maximum likelihood tree inferred from SRP54 sequences. Species codes represent the species names (Car - *M. carmabi*, For – *M. Formosa*, Dec – *M. decactis*) followed by sample number. Additional indices i.e. 1 or 2, represent alleles of the heterozygote samples. Bootstrap values (1000 replicate; >50%) are shown next to the branches.

From the three genetic markers studied for Madracis, the two nuclear introns (ATPSα and SRTP54) contained enough variation to allow phylogenetic inference. The mitochondrial nad5 contained minor genetic variation and was, as found also in other studies [13], [18], not suitable for comparisons within a group of closely related species. None of the inferred phylogenies contained monophyletic clades (Figure 2a and figure 3). In the ATPSα topological tree (figure 2a) we distinguished two clades. Clade I consisted of five *M. formosa* colonies and included one *M. decactis* colony. Clade II contained all *M. carmabi* colonies, two *M. decactis* colonies and one *M. formosa* colony. A phylogeny inferred from the SRP54 nuclear intron marker is shown in figure 3. The resolution of this phylogeny was relatively poor and all clades comprised a mixture of several species, a pattern found earlier by authors that used different genetic markers [19].

A molecular phylogenetic tree is shown in figure 2 together with a tree based on morphological distances. The dendrogram inferred from morphological data based on whole colony characteristics was better resolved than the one inferred from genetic samples. There is no full congruence between the morphological and genetic data sets despite the fact that almost all *M. carmabi* species are on the same clades in both trees. The same holds for *M. formosa*. The morphological tree shown in figure 2b contains three clades where each clade corresponds to one of the three *Madracis* species indicating a close match between the original species definitions based on corallite characteristics and differences in overall colony morphology. Only two exceptions were observed: the *M. decactis* group contained one *M. carmabi* colony (*Car436*) and the *M. carmabi* group contained one *M. formosa* colony (*For429*).

The degree of similarity between morphological and molecular phylogenies was measured using a Mantel test and the CADM (Congruence Among Distance Matrices) test. The results of these tests for morphological and genetics distances are shown in table 1. A direct comparison between genetic and morphological distance matrices (rows ATPSα and SRP54 in table 1) confirmed the absence of full congruence between morphological and genetic distance matrices. However, comparison of topological distances (defined by the number of branches between two leaves in a tree) were congruent (p<0.05) for the morphological tree and ATPSα based phylogeny accounting for 56% of the observed overlap.

**Table 1:** Mantel test and CADM (Congruence Among Distance Matrices) test results for morphological distance matrix and distance matrices inferred from ATPSα and SRP54 sequences.

Distance matrix type	Mantel t-value	Kendall’s coefficient W/p-value	CADM Mantel correlation/p-value
ATPSα	1.28	0.57/0.12	0.14/0.1
ATPSα T	1.57	**0.56/0.04**	0.13/0.06
SRP54	0.65	0.54/0.22	0.09/0.23
SRP54 T	0.84	0.55/0.13	0.11/0.13

T – topological distances. Number of permutations used for permutation test was 1000.

## Discussion

In this study we have, for the first time, quantitatively analyzed the degree of congruence between coral morphology at the colony level and molecular phylogenetics of the same colonies. Since traditional species classifications based on skeletal morphological characteristics rarely overlap with molecular phylogeny in scleractinian corals [6], this study explored new ways to quantify additional morphological traits based on whole colony morphology. The fact that groupings were found in our morphological analysis (based on branch thickness, branch spacing and branch length to thickness ratio) suggests that species-specific morphological traits were captured and measurable to some degree (Figure 2b). We do not find the same clustering in molecular phylogenies of the same individuals for all colonies. However, the molecular phylogeny (figure 2a) indicates complete separation between *M. formosa* (clade I) and *M. carmabi* (clade II) confirming the separation between the same species derived from whole colony morphological characteristics. The incongruence between morphological and genetically constructed phylogenies is not surprising. Coral phylogenies are usually complex and unresolved (e.g. due to reticulate evolution [13], or phenotypic plasticity [20], [21]). Therefore the incongruence between genetic and morphological classifications could reflect, for instance, different evolutionary trajectories of the species or the differential expression of key genes due to environmental pressure, rather than result from methodological errors [13]. The morphological tree showed separation between the three species, so the similarity shown in table 1 (row ATPSα T, W = 0.56) demonstrates the existence of at least some overlap between morphological and genetic data on the interspecific level. As stated above, several evolutionary mechanisms exist that can explain such incongruences. Recent speciation with incomplete lineage sorting andinterspecific introgressive hybridization are two evolutionary mechanisms often referred to in scleractinian phylogenetic studies to explain why molecular phylogenies do not match classical taxonomic classifications. For example, previous studies have shown that the genetic variation exhibited by some of the *Madracis* species can only be explained by hybridization events [19], [13].

The present analysis is useful to study interspecific variation. For example, consider the position of *Car436* sample in both trees (figure 2). This species, i.e., *M. carmabi,* is hypothesized to be a hybrid either between *M. formosa* and *M. decactis* [according to morphology, see 17] or between M. decactis and M. pharensis (according to genetic variation, see [13]). In our analysis *Car436* is the only *M. carmabi* sample that has a morphology different from the other *M. carmabi* samples in our data set. Its morphology (figure 1b) is closer to that *of M. decactis* than to a typical *M. carmabi* morphology. Surprisingly, one of the ATPSα alleles (*Car436-1,* Figure S1) of this sample is also genetically more similar to *M. decactis* than to the most *M. carmabi* species. In this case, this sample cannot be considered as misplaced in the phylogeny. Instead, morphological data supports the similarity of this sample to *M. decactis* species. Noteworthy is the fact that this is the only *M. carmabi* colony measured that has a heterozygous ATPSα genotype, with one of the alleles closely affiliated with *M. decactis* clades. This suggests that this may be a “carmabi-looking” colony that is strongly introgressed and has many *M. decactis* alleles. In the future, increasing the number of colonies analysed could help establishing to which degree this new method of measuring morphological distances can improve the interpretation of such mismatches between classical classification (based on corallite) and molecular phylogenies. It is also important to stress that in our previous study [9], which applied the same continuous quantification method of morphology, samples clustered separately from *M. decactis*. This same pattern was reported by molecular phylogenies using the same genetic markers applied here [13]. This highlights the applicability of the present method in separating species such as *M. mirabilis*, which unlike *M. decactis*, *M. formosa* and *M. carmabi*, do not overlap morphologically.

The comparison between phylogenetic trees and trees based on morphological characteristics can be used to identify species specific morphometric properties. In this analysis we are able to quantify the relations between genotype and phenotype (at the colony level). In future studies, this kind of analysis can be useful to find specific genetic characteristics that determine growth and form in a coral colony. In the paper by [22] it is suggested that the differences in colony morphologies in *Acropora* species is determined by a small number of genes. In a systematic quantitative comparison between colony morphologies and genetic data it might become possible to detect those genes. Such functional genes (involved in calcification, growth, etc) can then be used to construct phylogenies that can be compared to morphological data by applying the same method here described.

In the present study we analysed three Madracis species characterized by overlapping morphological and genetic variation. Besides pre-zygotic events (such as interspecific hybridization and consequent introgression, [13], [14], [15], [16], congruence between morphological and molecular phylogenies can also be affected by phenotypic plasticity [20], [21]. Corals are particularly prone to both levels of incongruence-generating events. We demonstrate that the method presented in this study can be useful in differentiating coral colonies from a genus characterised by a high degree of morphological plasticity and (likely) introgression.

## Materials and Methods

### Coral Colony Samples

Corals were collected from the fringing reefs of Curacao under permits issued by the Curacao Department of Environmental and Nature Management (Afdeling Milieu- en Natuurbeheer) of the Ministry of Health, Environment and Nature (Ministerie van Volksgezondheid, Milieu & Natuur). Coral specimens were collected by the CARMABI (Caribbean Research and Management of Biodiversity) research station, which has held a permit since 1976 for the collection of corals for scientific purposes. Corals were imported to the Netherlands under CITES permit AN001 held by CARMABI, and received under the University of Amsterdam CITES permit NL002. We obtained permission from CARMABI to use the collected coral colonies for research purpose at the University of Amsterdam.

Colonies of the three species, *i.e. M. carmabi* (n = 10), *M. decactis* (n = 10) and *M. formosa* (n = 7) were collected at depths between 6 m to 50 m on Curaçao (12° N, 69° W) [9]. We took DNA samples from five colonies of each species (total n = 15).The *Madracis* species in our dataset were classified according to morphological descriptions by Wells [23], [24], [17]. The number of septa is different between some of the species, i.e. *M. decactis* and *M. carmabi* have 10 septs while *M. formosa* has 8. CT scans of all colonies were made at a resolution of 0.33 mm × 0.33 mm × 1.50 mm per voxel. A data set for each colony contains between 45 and 765 image slices. 3D representations of all samples were reconstructed following the methods described in [8]. Four samples of such renderings are shown in figure 1.

### Genetic Data

Genomic DNA was extracted using the UltraClean Soil DNA kit (MoBio). Sequence variation was assessed for non-coding exon primed intron-crossing (EPIC) markers for three different genes: the mitochondrial DNA (mtDNA) subunit 5 of NADH ubiquinone oxidoreductase (nad5) [18] and the nuclear DNA (nDNA) ATP Synthetase Subunit α (ATPSα) and Signal Recognition Particle 54-kDa subunit (SRP54) [25]. A third nuclear intron, ATP Synthetase Subunit β (ATPSβ), was also included in preliminary surveys but due to the complete absence of sequence variation, this marker was discarded.

The nad5 intron was successfully amplified using the ND51b degenerate primer pair (NAD5_700F: 5′-YTG CCG GAT GCY ATG GAG-3′; NAD1_445R: 5′ARC CCA ATC GAA ACY TCA TAA CT-3′) of [18]. Nuclear introns were targeted with primer pairs described in [13]. ATPSα was amplified with primers ATPSαMadfor2 (5′-ACG AGA ACT TAT CAT TGG AGA CAG-3′) and ATPSαMadrev (5′-GGT GTC AAT CGC AAT AGC TG-3′). SRP54 was amplified with primers SRP54Madfor (5′-GAT AAA GTC AAT GAA CTG AAG C-3′) and SRP54Madrev2 (5′-TGG AAT TGT TCA TAC ATG TCT C-3′).

PCR protocols, PCR cycling conditions and denaturing gradient gel electrophoresis (DGGE) approach were applied as in [13]. DGGE profiles were characterized either by a single band (homozygote genotypes) or by quartet banding (heterozygote genotypes). In the last case, two of these bands corresponded to heteroduplexes, the result of re-annealing of heterogeneous DNA single strands during PCR [26]. All bands were excised, reamplified and reloaded on DGGE to evaluate band isolation. PCR products to be sequenced were purified using the QuickClean 5 M PCR Purification kit (Genscript). Sequencing was performed in forward and reverse directions by Macrogen Korea (http://dna.macrogen.com/eng/).

### Phylogenetic Analysis

The multiple sequence alignment was performed using Clustal W algorithm [27] in MEGA 5.0 [28]. For each sequenced region phylogenies (figures 3 and S1) were inferred with Maximum Likelihood method using MEGA 5.0 software. P-distance was used as genetic distance measure. Robustness of the nodes in figure 3 was assessed by non-parametric ML-bootstrap analysis (1000 pseudoreplicates) with random stepwise addition and nearest-neighbor interchange (NNI) branch swapping. The topological distances between the leaves in the ML trees in figure 2 were computed as cophenetic distances in these trees with all branch lengths equal to 1.

Some samples (colonies) in our data set were heterozygote for one or both nuclear intron markers. In order to standardize data comparison we duplicated all data for homozygote samples. All data comparisons were determined in separate for each allele and results averaged to provide a single data point. For instance, the genetic distance between two samples was computed as the average value between four inter-allele distance comparisons.

### Morphological Analysis

**Figure 4:**
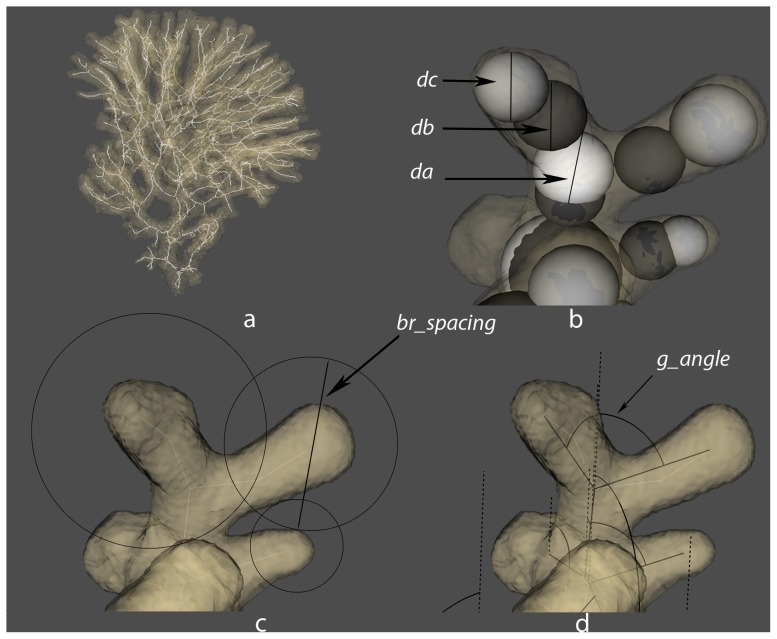
Morphometrics of branching coral colonies. a) Morphological skeleton generated from a volume of a CT scan of a Madracis colony, b) branch thickness, da – white sphere, db – black sphere, dc – gray sphere, c) branch spacing (br-spacing) and d) branching angle relative to the growth direction (g_angle).

Computed tomography (CT) scans of the collected coral colonies were measured using a morphometric method for complex-shaped branching objects [8]. To measure branch thickness a sphere is drawn centered at the medial axis of a branch. Since this sphere is bounded by the branch volume, the diameter of the sphere equals to the thickness of that branch. Therefore, branch thickness at the beginning of a branching point is defined as the diameter (*da*) of the white sphere in figure 4b. The diameter (*db*) of the black sphere, figure 4b, defines the branch thickness after branching. The diameter (*dc*) of the grey sphere located at the end point of a branch defines the thickness of a branch tip. Branching angle, (*b_angle*), is the angle between the medial axes of two connected branches. Branching angle relative to the growth direction, (*g_angle*), is measured between the positive y-axis and a branch, figure 4d. Branching rate, (*rb*), is the length of the branch before it splits. Branch spacing, (*br_spacing*), is equal to the radius of a sphere centred at the branch tip, which reaches the closest neighbouring branch (figure 4c). More detailed information about the algorithms used by the morphometric software can be found in [8].

### Morphological Phylogeny

To be able to compare molecular phylogenies with morphological data we constructed a morphological tree based on the normalized morphological distances between the coral colonies. These distances were computed between the morphological traits that describe the most variation in morphology. These traits *i.e.* branch thickness, branch spacing and branch length to thickness ratio, were identified using principal component analysis (see figure A.6. in [9]). A morphological distance matrix was then calculated using Euclidian distance in space that is defined by the main three morphological features. The morphological tree was inferred from a distance matrix using an average linkage clustering method.

### Congruence Test

To test congruence between genetic and morphological distances we used Mantel [29] and CADM (Congruence Among Distance Matrices) tests [30]. Both test are designed to compare distance matrices. Therefore, to calculate the degree of congruence between the topologies of morphological and genetic trees we have computed topological distance matrices from these trees.

## Supporting Information

Figure S1 **Phylogenetic tree inferred from ATPSα sequences using the Maximum Likelihood method based on the Tamura 3-parameter model.** Bootstrap values (1000 replicate; >50%) are shown next to the branches. Samples codes represent the species names (Car - *M. carmabi*, For – *M. Formosa*, Dec – *M. decactis*) followed by the sample number. Additional indices i.e. 1 or 2, represent alleles of the heterozygote samples.
(TIFF)
