# Supplementary figures and images for: Comparison between Colony Morphology and Molecular Phylogeny in the Caribbean Scleractinian Coral Genus Madracis
Source: PLoS One. 2013 Aug 14;8(8):e71287. doi: 10.1371/journal.pone.0071287 (PMC3743869; doi:10.1371/journal.pone.0071287)

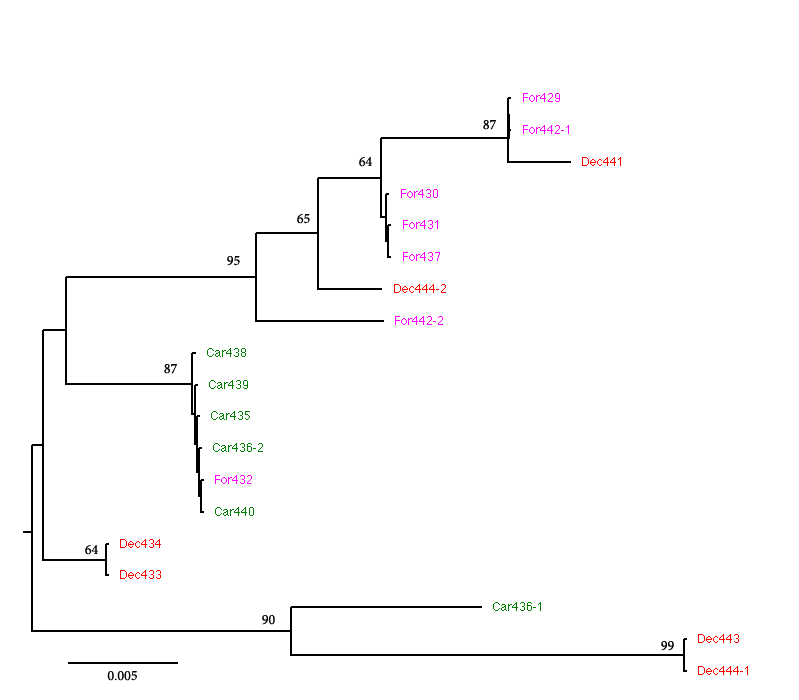

Supplement: Figure S1 — Phylogenetic tree inferred from ATPSα sequences using the Maximum Likelihood method based on the Tamura 3-parameter model. Bootstrap values (1000 replicate; >50%) are shown next to the branches. Samples codes represent the species names (Car - M. carmabi, For – M. Formosa, Dec – M. decactis) followed by the sample number. Additional indices i.e. 1 or 2, represent alleles of the heterozygote samples. (TIFF) [file pone.0071287.s001.tiff]
